# Mephistophles and the N. Y. Medical Journal

**Published:** 1885-08

**Authors:** 


### "MEPHISTOPHELES" AND THE NEW YORK MEDICAL JOURNAL.

The above caption is not intended to convey any suggestions of the "Devil and Tom Walker," or to make any parallel. Our esteemed contemporary is shocked because the *Denver Medical Times* has proposed to bestow upon us the *soubriquet* "Mephistopheles of Medical Journalism" and "envies the state of mind that enables us to reproduce the article with apparent satisfaction." The writer "wonders," also, "if Dr. Daniel ever read Faust," and "wonders if the *Denver Medical Times* realized with what sort of character it coupled the name of a man who was utterly free from guile." It is to enable our distinguished contemporary "to catch on to" the Mephistopheles business as applied to Medical Journalism, and to relieve his mind on the subject upon which, he says, he "wonders," by answering the inquiry as to ourself, and by giving the origin and application of the title, that we write this.

We would be doing violence to our very nature, were we to fail, in the beginning of a reply, to tender our most profound acknowledgments to our contemporary for the compliment (?) he pays our reading and general information, by the inquiry if we "ever read Faust?" While it is humiliating, it is due us, in order to be cleared of the intimation that we have not sense enough to see that the remarks of our Denver friend are susceptible of construction, quite the reverse of complimentary, to confess that we "*have* read Faust." Moreover, we have, a score of times, seen Von Huffland—the greatest living Mephistopheles—in that role, in the grand opera of Faust, and, also, the celebrated Dumestre; and we are not, therefore, in the state of blissful ignorance of that character the Journal affects to believe, or rather intimates.

The *Texas Courier Record*, of February, 1884, contained the following, which was the origin of the title, as applied to journalism:

"GAILLARD'S MEDICAL JOURNAL.—We have received the January number of this magnificent publication. We most cordially welcome it to our exchanges, for 'Gaillard's Journal' has now a world wide reputation, and is as standard in the world of medical literature as Dunglison's dictionary. To the constellation of lesser lights it is "a star of the first magnitude;" and wherever its light is shed, there is also warmth and comfort. The light, and the warmth, and the comfort may be due partly to the fiery red cover, which is not only pretty, but characteristic, but chiefly to the glow of professional zeal, and the scintillations of an intellect far above the ordinary. They say "black and red are the colors of Satan." We do not know if Dr. Gaillard ever thought of this, but he certainly is a very Mephistopheles in journalism; facinating, yet to be feared by his enemies, as much as he is admired by his friends. His success in journalism (this being his nineteenth year) has been something of a phenomenon, on which we do not know whether he or his readers are to be most congratulated.—*Texas Courier Record*, *Feb*. 1884.

Thus our New York brother will see that it *was* intended for compliment, and remembering that Mephistopheles frequently assumed the form of a facinating young man, (on which occasions he is said to have played the d—— with the gentle sex) we are proud to accept the title once born by the illustrious Gaillard, who, during his life, never questioned the propriety of it, in the sense in which it was used. The similarity of the journals in appearance may have suggested the idea, partially.

We regret to see, in the same paragraph from which we take the above, that the *New York Journal* accuses us of "misquoting its language and perverting its meaning." The spirit of resignation, and the air of injured innocence displayed in saying, "We have grown quite accustomed to it," reminds one of the experience of the eels, and touches us to the extent that we apologize, if we have done our neighbor injustice. We quoted from memory, but our readers, and his, if they desire to test the matter, can satisfy themselves if we are open to the accusation, by referring to the leading editorial of that Journal (*N. Y. Medical Journal*) for May 9, 1885, where they will find on page 526 the substance of our quotations, if not the identical words. We think the writer is ashamed of the harsh and inconsiderate expressions therein used, and would fain believe, and make believe, that we altogether misquoted and misapprehended his language. We hope our big brother will not put a chip on his shoulder and make faces at us; for we want to preserve amicable relations with him, and not being able to take a dare worth a cent, we are not ambitious to emulate the example, or share the fate of the traditional bull, whose pluck, being better than his judgment, led him to dispute the track with the locomotive under headway. *Savey* neighbor?

